# Mechanosensitive channels and bacterial cell wall integrity: does life end with a bang or a whimper?

**DOI:** 10.1098/rsif.2013.0850

**Published:** 2014-02-06

**Authors:** Marcel Reuter, Nicholas J. Hayward, Susan S. Black, Samantha Miller, David T. F. Dryden, Ian R. Booth

**Affiliations:** 1 School of Chemistry and COSMIC, University of Edinburgh, The King's Buildings, Edinburgh EH9 3JJ, UK; 2 School of Medical Sciences, Institute of Medical Sciences, University of Aberdeen, Foresterhill, Aberdeen AB25 2ZD, UK

**Keywords:** mechanosensitive channels, (bacterial) cell wall, bacterial stress response, optical tweezers, microfluidics, fluorescence-activated cell sorting

## Abstract

Mechanogated channels are fundamental components of bacterial cells that enable retention of physical integrity during extreme increases in cell turgor. Optical tweezers combined with microfluidics have been used to study the fate of individual *Escherichia coli* cells lacking such channels when subjected to a bursting stress caused by increased turgor. Fluorescence-activated cell sorting and electron microscopy complement these studies. These analyses show that lysis occurs with a high probability, but the precise path differs between individual cells. By monitoring the loss of cytoplasmic green fluorescent protein, we have determined that some cells release this protein but remain phase dark (granular) consistent with the retention of the majority of large proteins. By contrast, most cells suffer cataclysmic wall failure leading to loss of granularity but with the retention of DNA and overall cell shape (protein-depleted ghosts). The time span of these events induced by hypo-osmotic shock varies but is of the order of milliseconds. The data are interpreted in terms of the timing of mechanosensitive channel gating relative to osmotically induced water influx.

## 1. Introduction

Bacteria, for example *Escherichia coli*, maintain a positive turgor pressure estimated to range between 2 and 4 atm [1] and can survive transient changes when the turgor transiently exceeds 10–20 atm [2]. Cell integrity is maintained through the interplay between the peptidoglycan cell wall, which provides a constraining force, and mechanosensitive (MS) channels that modulate turgor. Turgor is generated by the selective accumulation of solutes up to molar concentrations, particularly when the cell is bathed in a high osmolarity medium [3]. A sudden lowering of the external osmolarity (also called ‘downshock’) precipitates rapid water movement into the cell such that turgor pressure can rise by greater than 10 atm in a few seconds [4–7]. The cell wall has some elasticity that accommodates the expansive pressure (R Phillips, M Bialecka-Fornal & HJ Lee, personal communication). Beveridge and co-workers [8] used atomic force microscopy (AFM) to predict that the peptidoglycan sacculus can expand by approximately 12% when subjected to a force of approximately 1 atm. However, to retain structural integrity during such transitions, MS channels gate on the millisecond timescale to effect large-scale release of solutes whenever the net outward pressure generates an increase in membrane tension [7–11]. Absolute parameters for the activation have been hard to establish in cells owing to the presence of the peptidoglycan cell wall and the linked outer membrane but, in isolated membrane patches, pressures as low as approximately 0.05 atm activate MS channels [10,12].

MS channels are ubiquitous in bacteria and consist of two main families, MscS and MscL [10,13]. MscL is a highly conserved, almost ubiquitous membrane protein [10]. MscS is also widespread, but in contrast to MscL, exists in many variant forms that are united by the presence of a ‘pore-forming’ domain close to the carboxy-terminal end of the protein [9,14]. MS channels gate in response to changes in the lateral tension generated in the cytoplasmic membrane by the change in turgor pressure across the membrane [2,10,15,16]. Organisms differ in the number of channels that they possess, some having only a limited complement of MS channels, whereas others, for example the γ-proteobacteria have up to six MscS homologues, in addition to MscL [10]. Work in *Escherichia coli* has established that MscS and MscL are central to the survival of rapid downshock, whereas the other MS channels, e.g. MscK and MscG in *E. coli*, modulate the osmotic (threshold) shock at which cell death occurs [9,17]. We have previously established that cell death is observed during downshock of mutants lacking MscS and MscL [9,18] and that this is accompanied by the release of protein and nucleic acid, from which we inferred loss of structural integrity. Subsequent studies on MS channel mutants in *Bacillus subtilis* [19] and *Lactococcus lactis* [20] have recorded that cell death arises upon downshock of such mutants, but studies of the mechanisms of cell death were not undertaken.

Structural integrity and cell shape are determined by the structure of the peptidoglycan [21–24]. In Gram-negative bacteria, there is usually a single layer of peptidoglycan covalently linked to the outer membrane through a variety of lipoproteins [21]. Peptidoglycan is a well-described polymer consisting of *N*-acetylglucosamine-*N*-acetylmuramic acid pentapeptide units (NAM-pp) units that are cross-linked through the formation of peptide cross-bridges [24,25]. While there have been different models for the structure of peptidoglycan [25], the currently accepted horizontal-layer model for *E. coli* is that the sugar chains are laid down around the circumference of the cell with the peptides forming cross-links in the direction of the longitudinal axis [24–30]. This simple picture omits many more complex features. First, the length of the glycan units is variable (9–30 NAG-NAM-pp units per sugar polymer) meaning that as many as 300 separate polyglycan units may be required to completely encircle the cell. Additionally, the glycans adopt a spiral format along the axis of the glycan chain such that each peptide emerges at a different angle with respect to the axis of the glycan chain, either 90° or 120° for the horizontal layer and scaffold models, respectively. Cross-linking to other NAG-NAM units to create a structurally integral wall is incomplete and variable with growth phase. Moreover, the cell must create breaks in the cross-bridges to allow new material to be inserted during cell elongation, but must also create ‘holes’ (greater than 70 Å diameter) to accommodate the large protein complexes, such as flagellae and secretion assemblies that span the cytoplasmic membrane and cell wall. Finally, some of the peptides are cross-linked with lipoproteins in the outer membrane, thus establishing a fixed connection between the two structures [21,22]. Recent work has augmented the biochemical view of the cell wall with approaches based on modelling and AFM [8,27–29,31,32]. In summary, the peptidoglycan is a highly dynamic, ‘disrupted’ mesh that contains a large number of lacunae of variable sizes that reflect the degree of cross-linking of the peptides and the variable length of the glycan chains. It is this structure that must both grow in the longitudinal direction to permit cell growth and simultaneously must resist the turgor pressure directed from the cytoplasm [26–28,33].

In this study, optical tweezers combined with microfluidics have been used to visualize single-cell lysis providing novel insights into the dynamics of cell death during hypo-osmotic shock in cells lacking the major MS channels: MscL, MscS and MscK [9]. These studies are supported by electron microscopy and by fluorescence-activated cell sorting (FACS) analysis of cell populations subjected to hypo-osmotic shock. The data show that individual cells suffer differing fates, but that the majority of cells lyse in a manner that generates a cell-shaped ghost that retains DNA and, owing to protein release, has lost granularity. Some cells, observed by optical tweezers, retain granularity but suffer transient membrane lesions that allow release of green fluorescent protein (GFP).

## 2. Material and methods

### 2.1. Strains and genetic manipulations

*Escherichia coli* FRAG1 (F^−^, *rha, gal, thi, lac*) and its mutant derivative *E. coli* MJF465 (FRAG1, *mscL::Cm; *Δ*mscS; mscK::Kan*) [9] were used throughout this study. *Escherichia coli* MJF465(DE3) was created using the Novagen DE3 lysogenization kit. Plasmid pET20-GFPuv was created using the GFP allele from pTYB1GFP (gift of Derek MacMillan, Department of Chemistry, University College, London, UK), which was amplified by the polymerase chain reaction, using 5′CCGGGACTTCACATATGAGTAAAGGAGAAGAAC3′ and 5′ATGCCTCGAGAAGCTTGAATTCTTAATGATGATGATGATGATGCTTGTACAGCTCGTCCATGCC3′ as primers, ligated into pET20 and the DNA sequence verified. Transformed cells were prepared using an MgCl_2_/CaCl_2_ protocol [34]. Antibiotics required for selection were carbenicillin, chloramphenicol and kanamycin (100, 25 and 50 µg ml^−1^, respectively).

### 2.2. Medium

Cells were grown in LB medium (per litre: 10 g tryptone, 5 g yeast extract and 5 g NaCl) or McIlvaine's citrate–phosphate buffer-based minimal medium [35]; 14 g l^−1^ agar was added for solid media. High osmolarity plates and solutions contained a further 0.5 M NaCl. The osmolality of the growth media was measured using a MicroOsmometer (VitechScientific Limited, Sussex, UK), following the manufacturer's instructions. For phase-contrast microscopy and optical tweezers manipulation, cells were grown to stationary phase, spread on LB agar plates containing 0.5 M NaCl and then incubated for 14 h ± 15 min at 37°C. Cells were harvested from the plates in high osmolarity medium. To prepare *E. coli* MJF465(DE3) pET20-GFPuv cells for epifluorescence microscopy and optical tweezers manipulation, exponential phase cultures were diluted 1/10 into LB + 0.5 M NaCl, grown until OD_600_ ∼ 0.1 when 0.1 mM IPTG was added to induce GFP expression. Cells were grown to OD_600_ ∼ 0.2–0.25 whereupon they were used.

### 2.3. Optical tweezers

The optical tweezers set-up has been described previously [36]. The laser traps were controlled through software written in LabView 6.1 (National Instruments) to change the *x*- and *y*-positions of the trap. The system was combined with epifluorescence imaging and built around a Nikon TE2000 microscope using a mercury lamp and a dichroic filter cube for GFP imaging containing for excitation a 450–490 bandpass filter, a 505 dichroic mirror and for emission a 520 longpass filter). Images were taken with a CCD camera (shutter time 50 ms, Gain 0; Dolphin 145-F, Applied Vision Technologies). Video imaging used HyperCam2 software, which captured images at 5 frames s^−1^.

### 2.4. Microfluidic devices

Microfluidic cells were fabricated from Perspex with two or three inlet channels meeting at an angle of 30° and one outlet channel (see electronic supplementary material, figure S1). These were milled to 0.07 ± 0.01 mm depth using a 0.4 mm diameter end mill. Channel width before and after the junction was 2 mm and 4 or 6 mm, respectively. Inlet and outlet holes (1 mm diameter) were connected to silicon tubing (bore 1.0 mm, wall 0.5 mm; 01-93-1407/05, Altec Products Ltd). The flowcell was sealed by gluing (Norland optical adhesive no. 61, Thorlabs) a cover glass (22 × 50 mm, Menzel-Glaeser) over the channels and exposing it for 20 min to UVA light (C-10P Chromato Vue Cabinet, Ultra-Violet Products Ltd). A KDS200 syringe pump was used in flow experiments (KD Scientific Inc.).

### 2.5. Single-cell experiments

Microfluidic devices were used in combination with optical tweezers to subject single cells to a hypo-osmotic shock from LB + 0.5 M NaCl into distilled water. A syringe was loaded with cell suspension and another with sterile distilled water. A flow rate between 20 and 40 µl h^−1^ per syringe was maintained, the maximum range at which cells stayed in the laser traps. The laser input power was maintained at 70 mW for trapping and manipulating cells, i.e. for moving them across the flow boundary. Two individually trapped cells could be observed simultaneously. Measurements lasted approximately 2 min allowing approximately 20–25 individual cell assays in 1 hour, i.e. giving data for 40–50 cells per experiment. A modified assay was used to counteract potential oxygen depletion in the microfluidic devices in which cells were grown overnight on LB agar plates containing 0.5 M NaCl, as previously, but harvested and suspended in minimal medium + 0.5 M NaCl. The third (middle) channel contained minimal medium + 0.5 M NaCl + 50 µM hydrogen peroxide where the *E. coli* cells resided for 30 s to recover before they were dragged into the lower channel containing sterile distilled water. The boundaries between the different streams were not sharp due to the relatively low flow rate and diffusion. We estimated that transfer of cells from high osmolarity into low osmolarity took between 5 and 10 s in our microfluidic devices. Thus, the cells experienced a gradual change in salt concentration during hypo-osmotic shock.

### 2.6. Image processing

Videos were cropped and reduced in frame number using ImageJ [37]. The same software was also used to read out fluorescence intensity values of single brightly fluorescing *E. coli* cells over the time course of a few minutes. For this purpose, a short macro was written.

### 2.7. Fluorescence-activated cell sorting

**Figure 1.**
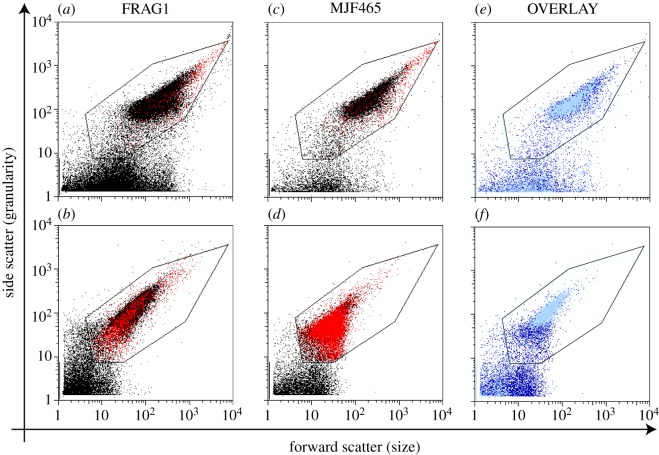
FACS analysis correlating cell size and cytoplasmic granularity (forward and side scatter, FS and SC) and uptake of propidium iodide (PI) into damaged cells that have retained the nucleoid. PI positive cells are coloured red. (*a*) Unshocked *E. coli* FRAG1 parental cells; (*b*) hypo-osmotically shocked *E. coli* FRAG1 cells; (*c*) unshocked *E. coli* MJF465 channel mutant cells; (*d*) hypo-osmotically shocked *E. coli* MJF465 cells. (*e*) and (*f*) overlay of data from control (*e*) and shock (*f*) samples (pale blue dots, FRAG1; dark blue dots, MJF465). In both cell populations, a significant number of particles with high FS and low SC were also observed in the high osmolarity samples and were found to be non-biological material arising from the buffer solutions (see the electronic supplementary material, figure S2). (*a–c*) Approximately 30 000 events are within the gate (see §2), but in (*d*), this falls to approximately 19 000, owing to cell lysis yielding material of the same size as background (see electronic supplementary material, figure S2a,b).

Cells were grown to an OD_650_ of 0.4 in LB, diluted to an OD_650_ of 0.05 in LB containing 0.5 M NaCl and grown to an OD_650_ of 0.4. These cells were diluted 1/20 into LB + 0.5 M NaCl or LB solutions that had been prefiltered (0.2 µm pore size) to remove small particles. The cells were incubated for 10 min at 37°C, collected and suspended in iso-osmotic McIlvaine's citrate–phosphate buffer minimal media [35]. The suspension was diluted 1/50 into iso-osmotic media containing 30 µM propidium iodide [38], incubated for 10 min and analysed on a FACSCalibur Flow Cytometer (Becton–Dickinson). Fifty thousand events were counted, and the data analysed using WinMDI (v. 2.9, open source software), FCS express (v. 3, De Novo Software) or FlowJo (v. 10, Tree Star, Inc.). The instrument was calibrated by addition of beads (1 μm and 2 μm; Invitrogen, non-fluorescent microspheres; cat no. F-13838). The positions of these beads with respect to forward scatter agrees well with published data for bacterial spores and cells measured by FACS [39]. The FACS gate for data analysis was set by the observed upper limit of ‘events’ recorded in buffer-only controls (see electronic supplementary material, figure S2), and the perimeter was established to contain the majority of ‘events’ observed with *E. coli* FRAG1 control incubations (figure 1*a*).

## 3. Results

### 3.1. Cell viability

As previously established, cells of *E. coli* MJF465 (lacking the three major MS channels MscS, MscK and MscL) grown to mid-exponential phase in LB containing 0.5 M NaCl and subjected to a 0.5 M NaCl hypo-osmotic shock (downshock; by dilution 20-fold into LB medium) exhibited considerable loss of viability. Under these conditions, 9.4% ± 4.4% (*n* = 3 replicates) of the mutant *E. coli* MJF465 cells survived, whereas, within experimental error, all of the parental *E. coli* FRAG1 cells retained viability (90% ± 8%). Similarly, immediately after dilution, the mutant suffered a greater loss of light scattering, the OD_650_ being approximately 12 ± 5% of that of the parental strain, *E. coli* FRAG1. This effect was investigated further using FACS analysis. The changes in cell parameters (forward and side scatter and propidium iodide staining) as a consequence of osmotic downshock were measured for the whole population (figure 1*a–f*). Prior to hypo-osmotic shock, *E. coli* FRAG1 and *E. coli* MJF465 exhibited similar distributions of side scatter (granularity) and forward scatter (size). In both cases, a few cells were stained with propidium iodide (PI^+^), which indicates a reduced capacity for maintaining an energized cell membrane [38] (see electronic supplementary material, figure S3). As expected for a growing culture, there is a range of cell sizes evident from the scatter (FS 4 × 10^1^−2 × 10^3^) [39] but relatively narrow distribution of granularity (figure 1*a,c*). After downshock, *E. coli* FRAG1 cells exhibited increased numbers of PI^+^ cells consistent with transient cell damage, because approximately 90% of cells exhibited cultural viability. By contrast, cells of *E. coli* MJF465 (lacking MscS, MscK and MscL) exhibited large shifts in both side and forward scatter after downshock and most ‘cells’ were PI^+^ (80 ± 9% for *E. coli* MJF465 compared with 7 ± 2% for the parent), which indicates that they have retained their nucleoid but lost the ability to energize their cytoplasmic membrane (figure 1*b,d*). When the data from shocked cells are overlaid for *E. coli* FRAG1 and *E. coli* MJF465, it is clear that the latter has undergone a more substantial change in cell shape and granularity (figure 1*e,f*). PI^+^ *E. coli* MJF465 exhibited a decreased size spectrum with a significant fraction of the particles smaller than cells (figure 1 and electronic supplementary material, figures S2 and S4), which is consistent with cell lysis. The observation of some cells that do not exhibit PI fluorescence is consistent with the observed survival of a few MJF465 cells. Electron microscopic imaging of cells after the hypo-osmotic shock (see electronic supplementary material, figure S5) *E. coli* FRAG1 cells showed mostly intact, granular, cells, whereas *E. coli* MJF465 cells showed mostly empty ‘ghost’ cells which have lost their granular contents.

### 3.2. Downshock of single cells observed with phase-contrast microscopy

Optical tweezers were used to transfer individual cells from a high (approx. 0.5 M NaCl) to low osmolarity solution in a microfluidic device [40] (see electronic supplementary material, figure S1). The cells in the high osmolarity solution were optically trapped and held in the solution without any contact with the surfaces of the microfluidic device. The trapping force turns the cell so that it is viewed along its long axis and appears as a dark disc in phase contrast. The microscope stage was moved, so that the traps dragged the cells into the low osmolarity solution.

Initial experiments failed to detect cell lysis when the mutant *E. coli* MJF465 cells were subjected to a 0.5 M NaCl downshock. Investigation revealed that the cells had become anaerobic during their time in the microfluidic device and this was found to prevent lysis. To overcome this, we inserted an additional high osmolarity channel to form a *Ψ*-shaped microfluidic device [40] (see electronic supplementary material, figure S1), containing small amounts of hydrogen peroxide (50 µM) in the middle inlet channel to ensure cell respiration. This concentration of hydrogen peroxide is non-lethal and is often added to anaerobic cell suspensions to re-initiate aerobic respiration [41] and to growing cells to elicit an adaptive response [42–44]. Using this approach combined with phase-contrast microscopy, individual cells were observed to exhibit unique lytic events when transferred from the high osmolarity to the low osmolarity channel. Both parent *E. coli* FRAG1 and mutant *E. coli* MJF465 cells exhibited a range of lytic events, but the proportion of each type of event was very much greater with the mutant strain, as expected (see figure captions for individual data).

**Figure 2.**
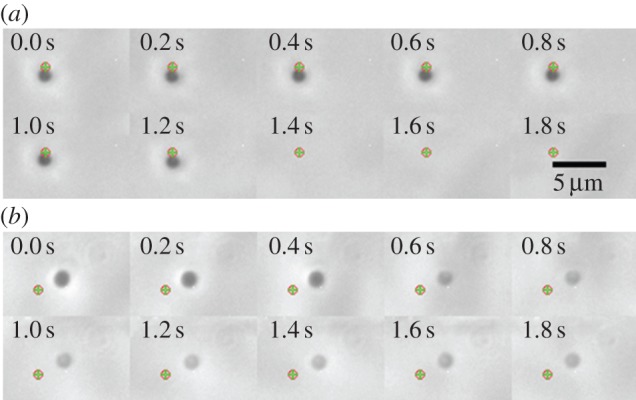
Phase-contrast microscopy time series for the transfer of an optically trapped *E. coli* MJF465 cell from LB + 0.5 M NaCl into distilled water via a third (middle) microfluidic channel containing 50 μM hydrogen peroxide. (*a*) A ‘burster’ cell that completely vanishes upon hypo-osmotic shock. No remnants could be observed in the entire field of view (fivefold larger). The full movie file is available as electronic supplementary material, movie S1. (*b*) A ‘leaker’ cell whose contrast decreases upon hypo-osmotic shock. Large cell wall ruptures (>20 nm) allow proteins to escape, which causes the image of the cell to fade. (The green cross and the red circle indicate the vicinity of the optical trap, for entire film see the electronic supplementary material, movie S2.) For this type of experiment, 40 *E. coli* MJF465 and 40 parent (*E. coli* FRAG1) cells were investigated. For MJF465, 17 cells lysed, 12 cells survived and the remaining 11 cells had an ambiguous outcome. By contrast, seven FRAG1 cells lysed, 19 cells survived and 14 cells had an ambiguous outcome.

Two distinct lytic events were observed. Some mutant *E. coli* MJF465 cells, ‘bursters’, disappeared in less than 0.2 s, the frame rate of the camera, with no detectable cell debris remaining in the optical trap (figure 2*a* and electronic supplementary material, movie S1). This was not due to simply losing the cell from the trap as in control experiments where the cell was deliberately released, the cell could be observed in subsequent frames being dragged away by the fluid flow until it disappeared from the field of view. Hence, these bursters have a half-life of less than 0.2 s. Other mutant cells, ‘leakers’, showed cell lysis in 1–3 s where a faint cell ghost remained in the trap (figure 2*b* and electronic supplementary material, movies S2 and S3) indicating a half-life of approximately 1 s. The formation of ghosts must be by loss of a substantial proportion of the ribosomes (approx. 20 nm diameter), which constitute at least 40% of total cell protein [45] and are a major contributor to the dark appearance of the cell in phase-contrast microscopy. Other proteins may also escape through these relatively large cell wall ruptures (at least 20 nm in diameter). Some mutant cells remained totally unchanged in appearance after the transition.

### 3.3. Downshock of single cells observed with fluorescence microscopy

**Figure 3.**
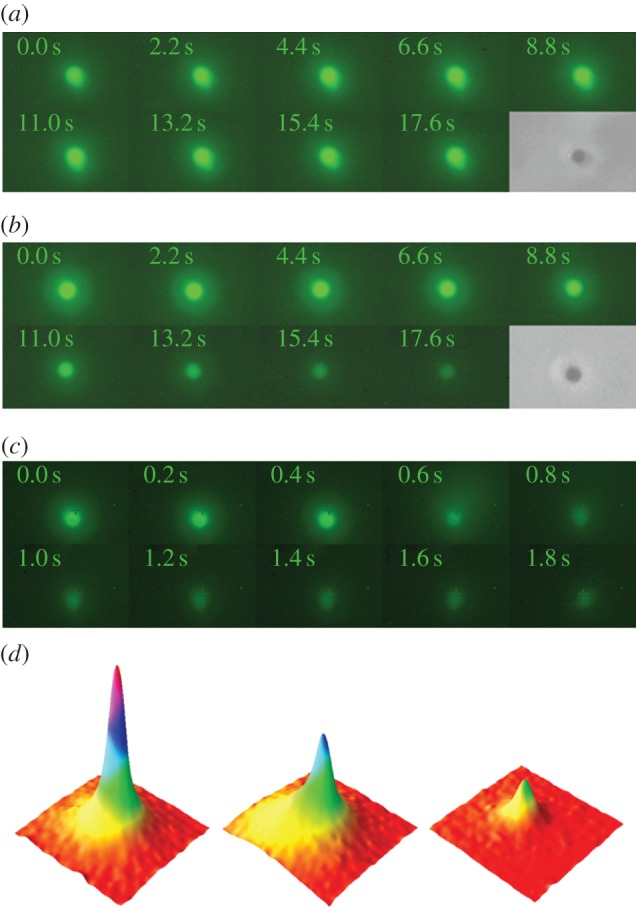
Fluorescence microscopy time series of optically trapped GFP-expressing *E. coli* MJF465(DE3) cells. (*a*) A control cell not subjected to downshock. Slow loss in GFP fluorescence occurs as a result of photobleaching. After complete GFP bleaching (300 s), the cell still appeared dark in phase-contrast mode (see electronic supplementary material, movie S4). (*b*) A cell subjected to hypo-osmotic downshock (LB + 0.5 M NaCl into distilled water via a third microfluidic channel containing 50 μM hydrogen peroxide). Owing to lesions, GFP leaks out of the cell resulting in a fast decrease in GFP fluorescence. After complete GFP leakage (30 s), the cell still appeared dark in phase-contrast mode (see electronic supplementary material, movie S5). The vast majority of cells (>95%) subjected to this treatment showed exactly this behaviour. For this experiment, 14 cells were subjected to hypo-osmotic shock and eight cells to control conditions, respectively. (*c*) A cell subjected to hypo-osmotic shock showing cell wall rupturing at 0.6 s. A burst of fluorescence could be detected moving away to the top right corner of this image (see electronic supplementary material, movie S6). This is an extremely rare event (<5%). (*d*) Individual frames from the movie in (*c*) were used to construct a three-dimensional plot where *x*- and *y*-axes are coordinates for each pixel and *z*-axis is the pixel intensity. Frames at *t* = 0 s, 1.80 s and 3.20 s, left to right, respectively, were plotted The plots are rotated 180° with respect to the frames presented in (*c*) to allow visualization of the GFP cloud, which would otherwise be hidden by the peak.

To obtain readily quantifiable data for cell lysis, we switched to fluorescence microscopy to observe release of GFP from cells upon downshock. GFP is expressed from a high copy number plasmid in the presence of IPTG, which induces T7 polymerase. Induction of the SOS response as a result of T7 polymerase expression [46] led to a mixture of cell filaments and cells of normal length; consequently, for this analysis we selected non-filamentous cells (less than 5 µm) with the optical traps. In iso-osmotic controls, such mutant cells exhibited bleaching of the intracellular GFP fluorescence on the 90–120 s timescale (figure 3*a* and electronic supplementary material, movie S4), which established the time limit for observations with shocked cells. Observation of over 30 mutant cells transferred into the low osmolarity solution clearly showed that several different processes were occurring. Generally, cells exhibited a rapid loss of GFP fluorescence on a 10–20 s timescale, which was four to six times faster than the observed bleaching (figure 3*a*). After GFP loss, phase-contrast microscopy confirmed that the cell was still retained in the optical trap and was phase dark, indicating that ribosomes and other major proteins had not been lost (figure 3*b* and electronic supplementary material, movie S6). Thus, the events observed in GFP-loaded cells are more subtle than the explosive ruptures observed by phase-contrast microscopy. During GFP leakage out of the cell, GFP was quickly flushed away by the fluid flow; thus, we clearly monitored the loss of intracellular GFP upon hypo-osmotic shock. A rare event (2/100 observations) was that GFP-expressing cells exhibited bursting upon hypo-osmotic shock (figure 3*c*). Such cells exhibited a plume of GFP that rapidly washed away in the flow device (see electronic supplementary material, movie S5).

**Figure 4.**
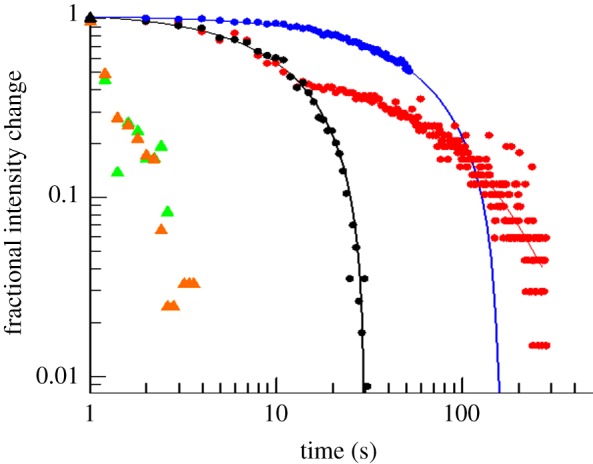
Fractional changes in intensity for the different classes of cellular events. Bursting of *E. coli* MJF465 cells with subsequent vanishing of all debris (black triangle) occurs in less than 200 ms (shown as a single point). Leakage of large material from mutant cells (green triangles) happens fast, on a timescale of 1 s. The bursting of a GFP-expressing *E. coli* MJF465(DE3) cell (orange triangles) was rare and occurred on the 1 s timescale. GFP leakage (black circles) and GFP photobleaching (blue circles) from MJF465(DE3) cells were characterized by a mono-exponential decay function. Individual traces for MJF465(DE3) cells that were hypo-osmotically shocked without hydrogen peroxide support (red circles) exhibited an initial rapid decrease in GFP fluorescence (leakage from cell) followed by a slower decay, consistent with fluorescence bleaching of the retained GFP. Out of 14 shocked cells, five cells displayed this biphasic behaviour, whereas the remaining shocked cells behaved like control cells (nine cells tested), and thus did not release GFP.

The fractional change in signal obtained for the various categories of hypo-osmotic shock-induced events exhibited limited cell-to-cell variation (figure 4). GFP fluorescence bleaching in cells not subjected to downshock (figure 3*a* and figure 4 blue data), showed a simple exponential decay (mono-exponential decay law *I_t_* = *I*_0_exp(−*kt*), *I*_t_ intensity at time *t*, *k* rate constant) corresponding to a rate constant of 0.010 ± 0.002 s^−1^. The decrease of the intracellular fluorescence in shocked cells (figure 3*b* and figure 4 black data) could also be fitted to a first-order decay process with a rate constant of 0.043 ± 0.004 s^−1^ i.e. approximately fourfold faster. When we performed the same experiments in the two-channel microfluidic device (without the H_2_O_2_ channel) a proportion of mutant cells showed an initial leakage of GFP on the approximately 10–20 s timescale followed by a second phase in which GFP fluorescence faded at the same rate as bleaching in control cells (figure 4, red curve). The rate constant for the initial event was the same as for the cells exhibiting complete leakage of the GFP and the rate constant for the second slow process was identical to that for photobleaching. These data would be consistent with an initial membrane rupture resealing such that only partial loss of the intracellular GFP occurs.

## 4. Discussion

We have previously established that *E. coli* cells lacking the major MS channels, MscS, MscK and MscL, die upon severe hypo-osmotic shock [9]. In this study, we have sought to define some of the critical events that are concurrent with loss of viability. Our data show that cell death leads to the formation of cell-shaped ghosts and that these ghosts retain their nucleoid (PI^+^) despite the loss of granularity associated with cytoplasmic protein (figure 1). Single-cell analysis points to multiple pathways leading to cell lysis with some cells ‘leaking’ and others ‘bursting’ over a short time period, usually less than 2–3 s (figures 2–4), whereas others appear to retain their integrity over the assay period (up to 2 min). In these single-cell experiments, only 17 cells lysed, with a further 11 being scored ambiguous and 12 cells not lysing at all (figure 2). The failure of some cells to burst accords with previous data that consistently find that approximately 5–10% of mutant cells survive the downshock and the observation that protein release (as a measure of cell lysis) continues for up to 20 min after shock [9] (S Black, S Miller & IR Booth 2013, unpublished data), which may indicate a fraction of cells that burst as the cells start to reinitiate growth [9,18]. We have previously described survivors as physiological variants to indicate that their subsequent culture and submission to hypo-osmotic shock recapitulates the original experiment [9]. There are many factors that might assist such cells in survival. *E. coli*, in common with many other bacteria, possesses other MS channels that appear to be limited in their contribution to survival by the level of their expression in the cell [47] but that modulate the threshold hypo-osmotic shock at which cell death occurs [17]. Although systematic studies of channel abundance are in their infancy [48], there are good reasons to expect significant cell-to-cell variation in the expression of individual proteins [49,50]. It follows that a source of transient protection against rapid, severe, hypo-osmotic shock in the mutant cells would be variation in expression of alternative channels, YbdG, YnaI, YbiO and YjeP [47]. We have previously established that MscG (YbdG) exerts a small, but measurable effect on cell survival in the genetic background lacking MscS, MscK and MscL and that this is manifested as a change in the threshold in salt concentration required during downshock to effect cell death [17]. Studies in which hypo-osmotic shock was applied at a controlled rate have found that the identity of the channel(s) expressed by cells is a critical determinant of survival (M Bialecka-Fornal, HJ Lee & R Phillips 2013, personal communication). Other factors may include the degree of cross-linking of the peptidoglycan, such that there are few or even no large tesserae that can become ripped and torn upon downshock. However, the analytical power to determine the frequency of wall structure variation has not yet been developed, although modelling approaches are beginning to be established [28,30]. We have noted an additional effect here that may be specific to this study. During cell storage, prior to their subjection to the optical trapping, the cells may become anaerobic and this can be alleviated by the supply of non-lethal, low concentrations of hydrogen peroxide [41,43] immediately prior to transfer to the low osmolarity channel. Respiration will allow the regeneration of the membrane potential and this may lead to restoration of higher potassium pools [51], which are essential for high turgor.

Hypo-osmotic shock leads to rapid entry of water into the cell down the osmotic gradient. This process is likely to be extremely rapid taking milliseconds to seconds rather than minutes [4–7,52]. Beveridge and co-workers [8] measured the forces that could drive the stretching of the peptidoglycan. Note, however, that these measurements [8] were made on relaxed isolated sacculi rather than growing cells. In the latter, the peptidoglycan is already stretched and may not have the capacity for expansion without ‘tearing’ of the fabric of the wall. One can calculate the potential expansion associated with water influx by treating *E. coli* as an open-ended cylinder that can stretch in its long dimension without modification of the radius. Such an approximation is justified in this instance, because the hemispherical poles are believed to exhibit low turnover and may represent highly cross-linked peptidoglycan that is not susceptible to stretching [53]. Previous work suggests that the peptide bonds are oriented in the direction of the length of the cell [27] and have the capacity to stretch, but the circumferentially arranged sugar chains are more rigid. As an example, 1 atm force exerted on the peptidoglycan could expand the area by up to approximately 12% [8], which would cause a 12% increase in cell volume. Such an expansion would require approximately 10^9^ water molecules to enter the cell (see the electronic supplementary material, note S1). The rate constants for water movement have only rarely been measured in bacterial cells. It has been suggested that the aquaporin may increase permeability by 30-fold [4], but others have failed to observe effects of the absence of the aquaporin on cell shrinkage in response to hyperosmotic shock [5]. However, from separate studies, it is clear that *E. coli* cells can lose 30% of their water content (approx. 10^10^ water molecules) in approximately 1 s [5,6]. Consequently, it follows that water influx to generate an increase in turgor of approximately 1 atm may take as little as 100 ms, but at the most 1 s. A 20-fold dilution of cells adapted to high osmolarity (LB + 0.5 M NaCl) into LB would constitute a potential increase in turgor of approximately 20 atm. Thus, the pressure transitions in a bacterial cell undergoing hypo-osmotic stress can be huge and very rapid, thus requiring MS channels to gate on the millisecond timescale. Indeed, these calculations fit with light scattering measurements using a stopped flow apparatus in which Sukharev and co-workers [7] measured osmotically induced swelling for 30–50 ms after mixing with low osmolarity medium.

The measurements made here with optical tweezers make an initial definition of the timescales for the failure of the cell wall when cells either lack MS channels or fail to gate them. Thus, in GFP-expressing cells, the release of fluorescence is essentially complete (in those cells that leak rather than burst) in 20–30 s (figure 4). Observations by phase-contrast microscopy of trapped cells show lytic (bursting) events take place in 1–2 s (figure 2). Phase-contrast microscopy limits observations to bursting cells, because leaking can only be easily discerned in cells tagged with a fluorescent protein. We have previously established that the change in salt concentration required to elicit gating of the channels in *E. coli* cells is just slightly smaller than that required to cause significant cell death in channel-free cells [9]. Combining these two analyses, a picture emerges in which the sensitivity of the channels to membrane tension induced by hypo-osmotic shock and the robustness of the cell wall interplay to provide the protection mechanism which is a 1–2 s window in which high turgor pressure must be relieved to prevent cell death. Others have shown that there is a definite rate contribution, i.e. the rate at which osmotic pressure changes influences the fate of the cells [48] (M Bialecka-Fornal, HJ Lee & R Phillips 2013, personal communication). Clearly, modulating the rate of change of the external osmolarity modifies the rate of water entry into the cells and expands the time window within which channels may gate to alleviate the stress before the cell wall fabric is torn. Moreover, this also provides time for the minor channels to contribute to solute release despite their low abundance [47] and accords with the observed changes in the salt concentration required to cause cell death in cells depleted of multiple channels [9,17]. Moreover, it is a factor in the well-documented effects of channel abundance on cell survival [18,54].

Do cells die with a ‘bang or a whimper’? The diameter of GFP is 3–4 nm compared with the approximately 20 nm diameter of the ribosome and their cross-sectional areas differ by approximately 40-fold. Measurements indicate that the cell wall is generally only permeable to proteins of less than 100 kDa with holes of only approximately 6 nm diameter [23]. However, the cell wall can stretch considerably [8,32,33], so the holes are able to expand. In addition, variability in the number and location of peptide cross-links may also produce larger holes [24,25,28,30]. In the horizontal-layer model [24,25,30], we can calculate that the two smallest well-defined pore sizes are approximately 6 and approximately 15 nm in diameter (see electronic supplementary material, note S2). These holes are large enough to let GFP escape once ruptures are made in both the cytoplasmic membrane to release the protein to the periplasm and in the outer membrane for release into the surrounding medium. In studies with sublethal concentrations of vancomycin, *E. coli* extrude small blebs (‘bulges’) on the outer surface that have been used in AFM studies to estimate turgor pressure [31]. These blebs can be accessed by GFP from the cytoplasm, and thus might be the precursor to full cell lysis. Bursting of such blebs could release the cytoplasmic proteins giving rise to both ‘leakers’ and ‘bursters’. The bursters must possess either larger rips or tears that are sustained for longer periods before the membrane reseals. However, if the bleb is either small and/or resolves by resealing it could give rise to the phase dark cells that predominantly release small proteins, for example GFP (‘leakers’). It has been calculated that the majority of the GFP in the cell can leave through a small perforation in less than 6 s, which sets the limit for sealing of the bleb ([55]; NS Wingreen 2013, personal communication). Thus, we propose that there are multiple fates for cells that are influenced by, first, the activity of the residual MS channels in strain *E. coli* MJF465, second, the patterns of cell wall cross-linking at the time of downshock and, lastly, the ability of the membrane to reseal without substantial loss of protein, such that cell viability is only transiently impaired.

In conclusion, our data show that the cell wall and membranes of some cells fail catastrophically (death with a bang) while we infer from GFP leakage that some other cells develop approximately 10 nm-scale lesions, in both the inner and outer membrane, sufficient to allow the escape of macromolecules via diffusion (death with a whimper).
